# A review of the barriers to using Patient‐Reported Outcomes (PROs) and Patient‐Reported Outcome Measures (PROMs) in routine cancer care

**DOI:** 10.1002/jmrs.421

**Published:** 2020-08-19

**Authors:** Hanh Nguyen, Phyllis Butow, Haryana Dhillon, Puma Sundaresan

**Affiliations:** ^1^ Western Sydney Local Health District Radiation Oncology Network Sydney Australia; ^2^ Centre for Medical Psychology & Evidence‐based Decision‐making The University of Sydney Sydney Australia; ^3^ Sydney Medical School The University of Sydney Sydney Australia

**Keywords:** patient‐reported outcomes, patient‐reported outcome measures, barriers, cancer, oncology

## Abstract

**Introduction:**

Patient‐reported outcomes (PROs) are direct reports from patients about the status of their health condition without amendment or interpretation by others. Patient‐reported outcome measures (PROMs) are the tools used to measure PROs; they are usually validated questionnaires patients complete by self‐assessing their health status. Whilst the benefits of using PROs and PROMs to guide real‐time patient care are well established, they have not been adopted by many oncology institutions worldwide. This literature review aimed to examine the barriers associated with using PROs and PROMs in routine oncology care.

**Methods:**

A literature search was conducted across EMBASE, Medline and CINAHL databases. Studies detailing barriers to routine PRO use for real‐time patient care were included; those focusing on PRO collection in the research setting were excluded.

**Results:**

Of 1165 records captured, 14 studies informed this review. At the patient level, patient time, incapacity and difficulty using electronic devices to complete PROMs were prominent barriers. At the health professional level, major barriers included health professionals’ lack of time and knowledge to meaningfully interpret and integrate PRO data into their clinical practice and the inability for PRO data to be acted upon. Prominent barriers at the service level included difficulties integrating PROs and PROMs into clinical workflows and inadequate information technology (IT) infrastructures for easy PRO collection.

**Conclusion:**

This review has outlined potential barriers to routine PRO use in the oncology setting. Such barriers should be considered when implementing PROs into routine clinical practice.

## Graphical abstract

Patient‐reported outcomes (PROs) are direct reports from patients about the status of their health condition without amendment or interpretation by others. This review article outlines potential barriers to routine PRO use in the oncology setting. Such barriers should be considered when implementing PROs into routine clinical practice.

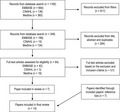

## Introduction

Survival, time to disease progression and clinician‐rated treatment toxicities have traditionally been the measures of patient outcomes in oncology.^1^ More recently, there has been increasing recognition that for true patient‐centred, quality healthcare to be delivered, knowing patient function and quality of life (QoL) outcomes is pivotal.^1^, ^2^ However, such outcomes are often inaccurately assessed by health professionals (HPs) and poorly captured by medical procedures and tests, highlighting the need for patient involvement in reporting their outcomes.^3^, ^4^, ^5^

Patient‐reported outcomes (PROs) are direct reports from patients about their health status (e.g. swallowing function during radiation therapy) without interpretation by others.^6^ Patient‐reported outcome measures (PROMs) are the tools used to measure PROs; they are usually validated questionnaires patients complete by self‐assessing their health status.^7^ Domains assessed by PROMs may include, but are not limited to, the patients’ physical, emotional, psychosocial well‐being and overall quality of life (QoL).^7^, ^8^ PROs and PROMs should not be confused with patient‐reported experiences (PREs) or patient‐reported experience measures (PREMs) which relate to patients’ perceptions of and/or satisfaction with the care they received.^7^

PROMs have historically been used solely in the research setting. The PRO data collected, alongside disease‐specific and toxicity outcomes, were used to compare different treatment interventions at a cohort level.^7^, ^8^, ^9^ In recent years, however, due to the reported benefits of routine PRO use, there has been widespread advocacy for the inclusion of PROs alongside standard clinical assessments by HPs to guide real‐time, individual patient care.^9^, ^10^, ^11^, ^12^

Routinely using PROMs empowers patients to actively participate in their health care, facilitates early detection and monitoring of patient symptoms, and enables HPs to better understand and act on patients’ needs.^8^, ^12^, ^13^, ^14^, ^15^, ^16^, ^17^, ^18^ PRO data also foster patient‐to‐HP communication by enabling HPs to routinely raise and review specific issues with their patients and allowing patients to elaborate on their symptoms and how they function in their daily lives.^14^, ^15^, ^16^, ^17^, ^18^, ^19^ Routine PROM use has also been shown to improve disease and treatment outcomes. A randomised control trial comparing chemotherapy patients who participated in PRO collection, against those who did not, showed that those in the intervention arm had significantly fewer emergency room visits, greater improvement in their health‐related quality of life (HRQL), better ability to tolerate treatment and a significant increase in overall survival (31.2 vs 25 months respectively).^19^, ^20^ Capturing population‐level PRO data systematically over time can also improve the safety and quality of healthcare delivery. For example, HPs and health services can use PRO data to monitor and identify gaps in performance, benchmark their patients’ outcomes against other services and guide policy and procedures related to service delivery.^21^

How PROs are collected and used in routine clinical practice is dependent on various factors such as the patient population, department workflows and available resources.^22^ Though traditionally collected on paper, it is increasingly common for PROs to be administered electronically using touch‐screen devices in clinic waiting rooms or on patients’ own devices.^23^, ^24^ Other considerations necessary include the PROMs used (e.g. disease‐ or treatment‐specific or general), the frequency and follow‐up period of PRO collection, the clinical pathways and actions triggered based on PROM scores and the electronic PRO collection system used which, in turn, can affect the way PRO data is collected, stored and displayed.^22^, ^25^

Given its many potential benefits, there is widespread interest to collect PROs using PROMs to guide routine patient care. However, successful and sustainable adoption of such new practices needs to be compatible with stakeholder needs and values, minimising the burden on them, understanding the barriers associated with routine PRO use is crucial.^14^, ^26^ The aim of this review was to examine the barriers associated with routine PRO and PROM use in the oncology context.

## Methods

**Figure 1:**
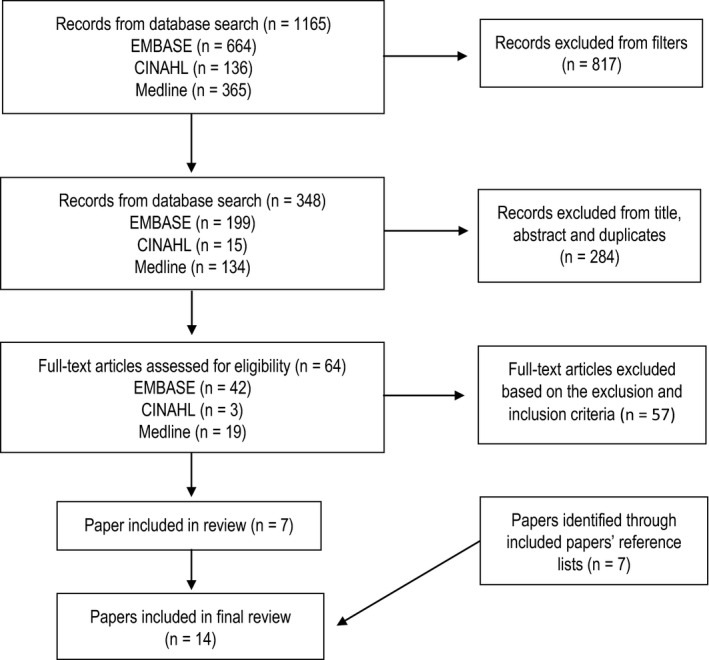
Process adopted for study selection.

A literature review was conducted across EMBASE, Medline and CINAHL databases in September 2019 (Fig. 1). Search terms, combined with BOOLEAN operators “OR” and “AND”, were as follows: [“patient reported outcomes” OR “patient reported outcome measures”] AND [“oncology” OR “cancer”]. The search period was from April 2013 to September 2019 inclusive. Results were filtered to full‐text English articles. Editorials were excluded as were conference abstracts. Any reviews published in this time period were used to extract original studies that met eligibility criteria whilst the review papers themselves were excluded. Studies focusing on routine PRO use for real‐time patient care were included whilst articles focusing on the collection of PROs for research purposes (e.g. to compare different interventions) were excluded. No restrictions were made on the method of PRO collection, the type of PROMs used, the study population and the study design.

Retrieved studies were checked for duplicates, and titles and abstracts were screened against the eligibility criteria by one reviewer (HN). A second reviewer (PS) screened 10% of titles and abstracts selected at random. If all criteria were met, or relevance was ambiguous, full‐text papers were obtained. Any disagreements between the reviewers were resolved through team discussion. Barriers to PRO use in the included papers were tabulated with each studies’ characteristics then extracted by the first reviewer (HN) and categorised into patient‐level, HP‐level or service‐level barriers for analysis and elucidation. A second reviewer (PS) independently completed data extraction for a random 10% of included studies to check for consistency. No disparities were found between reviewers.

## Results

Search terms captured 1165 titles across EMBASE, Medline and CINAHL electronic databases. Filters reduced results to 348 titles. After removing duplicate studies and eliminating articles based on title and abstract, full text of the remaining 64 was reviewed against the inclusion and exclusion criteria. One paper was added after examining the included papers’ reference lists. Overall, eight studies were retained for this review (Table 1).^10^, ^12^, ^14^, ^18^, ^26^, ^27^, ^28^, ^29^, ^30^, ^31^, ^32^, ^33^, ^34^, ^35^ Barriers to PRO use, as identified in these studies, were extracted and categorised into patient‐level, HP‐level or service‐level barriers (Table 2, 3, 4).

**Table 1:** Characteristics of included studies.

Author	Study design	Population	PROM Characteristics	Barriers identified
Hughes et al., 2004 ^29^	Qualitative semi‐structured interviews	Patients (n = 3) and nurses (n = 13) in UK palliative care setting	‐ Paper‐based PROMs: 6‐month experience ‐ PROMs (10 items): POS (physical, psychological and spiritual domains of life)	‐ Time constraints ‐ Staff busy work loads ‐ Some PROM issues identified could not be clinically addressed ‐ HP reluctance to recruit patients (e.g. patient distressed/unwell, need for informed consent, HP lack of confidence/competence) ‐ Intrusion on patient personal space
Basch et al., 2005 ^30^	Quantitative cross‐sectional survey	Patients (n = 74) undergoing chemotherapy for gynaecological cancer in US cancer centre	Web‐based (STAR) PROM collection: 6 months experience ‐ PROMs (13 items): adapted CTCAE, adapted ECOG performance status assessment, EQ‐5D	‐ Technical difficulties completing PROMs ‐ Patient inconvenience ‐ Liability issues
Kanatas et al., 2009 ^31^	Quantitative cross‐sectional survey	Members of British Association of Head and Neck Oncologists (n = 106)	‐ Unspecified mode of collection. ‐ PROMs: HRQOL questionaries including EORTC, FACT, UW‐QOL	‐ Time constraints ‐ HP difficulty analysing PROM data ‐ HP forgetfulness to distribute PROMs ‐ HP perceived lack of value added to patient clinical management ‐ Patient compliance ‐ Lack of resources for PROM collection
Snyder et al., 2010 ^32^	Qualitative semi‐structured telephone interviews	Breast and prostate cancer patients (n = 41) and doctors (n = 15) in US cancer centre	Nil (pre‐PROM implementation interviews)	‐ Time constraints ‐ Patient perceived irrelevance of certain PROM questionnaires ‐ Patient perception that PROMs may hinder the HP–patient relationship ‐ Burden upon patients to complete PROMs
Daveson et al., 2012 ^33^	Quantitative cross‐sectional survey	HPs (n = 392); doctors (n = 196); nurses (n = 104) in palliative care in Europe and Africa	Unspecified	‐ Time constraints ‐ Lack of training on PROM tools
Snyder et al., 2013 ^34^	Qualitative cross‐sectional interviews and quantitative surveys	HPs (n = 11), breast and prostate patients (n = 47) in US cancer centre.	‐ Web‐based (PatientViewpoint) PROM collection; feasibility phase ‐ PROM: all patients (physical, function, pain interference, social role satisfaction, fatigue, anxiety, depression), breast patients (EORTC BR23), prostate patients (EPIC short form)	‐ PROMs identified issues already known to HPs ‐ Patient perception that intervention may be impersonal ‐ Patient perception that HPs’ may not review PROM data ‐ Patient technical difficulties ‐ System technical issues (email notification issues, results not synchronising with EMR) ‐ Time constraints ‐ Patients sick/unwell
Judson et la., 2013 ^35^	Qualitative patient self‐reports	Patients (n = 286) undergoing chemotherapy at US cancer centre	‐ Web‐based (STAR) PROMs: 12‐month experience ‐ PROMs: EuroQoL EQ‐5D, CTCAE (pain, fatigue, nausea, vomiting, constipation, diarrhoea, appetite loss), performance status	‐ Patient forgetfulness ‐ Patient too busy/did not feel like reporting ‐ Patient sick/unwell
Hubbard et al., 2014 ^18^	Quantitative and qualitative cross‐sectional survey	HPs (n = 44): oncologists, oncology fellow, physician assistant, nurse in solid tumour oncology practice in the US	‐ Paper‐based PROM; 18‐month experience ‐ PROM (n = 3); pain, fatigue and overall QOL measured on a 0‐10 scale	‐ Unclear clinical pathways for actioning PROMs
Schepers et al., 2016 ^26^	Quantitative cross‐sectional survey	Paediatric HPs (n = 352): 52 countries worldwide	Unspecified	‐ Time constraints ‐ Insufficient staff to address issues ‐ Logistical problems ‐ Lack of financial resources ‐ PROMs not fitting into clinical workflows
Trautmann et al., 2016 ^10^	Qualitative non‐directed, narrative group interviews	HPs (3 nurses, 2 physicians) in a German cancer centre.	‐ Electronic PROMs; 6‐month experience ‐ PROM (79 items): EORTC QLQ‐C30, Distress Thermometer, HSI, Short‐Form MNA, BPI, Karnofsky index, Control Preference Scale	‐ Time for patients to complete PROMs pre‐consultation ‐ PROMs irrelevant to patient situation ‐ Lack of PROM response options ‐ HP lack of knowledge on PROM data
Baeksted et al., 2017 ^27^	Qualitative semi‐structured interviews	Oncologists (n = 5) and castration‐resistant metastatic prostate cancer patients (n = 4) in a Danish hospital	‐ Electronic PROMs (AmbuFlex); 3‐month experience ‐ PROMs (41 items): PRO‐CTCAE	‐ Patient late arrival to clinic; no time to answer PROMs ‐ Patient difficulty using PRO collection system ‐ Patient too ill to complete PROMs ‐ HP lack of knowledge on content/aim of PRO collection system ‐ HP lack of knowledge on PRO use ‐ HP inconvenience logging into another system ‐ Lack of pictures and graphs of patient symptoms. ‐ Lack of guidelines on PRO use ‐ Patient mis‐estimation of their symptom severity
Girgis et al., 2017 ^14^	Qualitative cross‐sectional survey, cognitive interviews and evaluation interviews	Oncology HPs (evaluation interviews n = 5) and patients (cognitive interview n = 10, survey n = 28, evaluation interviews n = 14) in an Australian hospital	‐ Electronic PROMs (PROMPT‐Care); 3‐month experience ‐ PROMs (67 items): Distress Thermometer, Edmonton Symptom Assessment Scale, SCNS‐ST9	‐ Patient difficulty recalling their symptoms ‐ Lack of opportunity for patients to discuss PRO data with HPs ‐ Patient responses not directly related to their cancer care ‐ Unresolvable identified issues regardless of information/support provided ‐ Inability for staff to review and address all issues in a single clinical consult. ‐ Increase clinical workloads and consultation times ‐ PROMs highlighted issues already known to the clinical team ‐ HP difficulty navigating through PRO collection system.
Duman‐Lubberding et al., 2017 ^12^	Qualitative semi‐structured interviews	Surgeons (n = 6) and HNC patients who no longer, or have never, participated in PRO collection (n = 71)	‐ Electronic PROMs (OncoQuest); 5‐year experience ‐ PROMs (79 items) ‐ EORTC QLQ‐C30 and QLQ‐H&N35 questionnaires, HADS	‐ Inadequate explanations to patients on PROs and PROMs ‐ Lack of feedback from HPs to patients on PRO data ‐ Time for patients to complete PROMs ‐ Value of PRO collection unclear to patients ‐ Delayed referrals to supportive care ‐ Identification of unsolvable problems
Wang et al., 2018 ^28^	Quantitative surveys and qualitative assessments	Chemotherapy patients (n = unknown) in cancer centre in US.	‐ Electronic PROMs ‐ PROMs (32 items): Three‐level version of the EQ‐5D‐3L PRO‐CTCAE	‐ Inconvenience and time to complete PROMs ‐ Patient too unwell to complete PROMs

PROM, patient‐reported outcome measures; HP, health professionals; POS, Palliative care Outcome Scale; CTCAE, Common Terminology Criteria for Adverse Events; ECOG, Eastern Cooperative Oncology Group; EQ‐5D, EuroQol‐5D; HRQOL, Health‐Related Quality of Life Questionnaires; EORTC, European Organisation for Research and Treatment of Cancer; FACT, Functional Assessment of Cancer Therapy; UW‐QOL, University of Washington Quality of Life Questionnaire; EPIC, Expanded Prostate Cancer Index Composite; STAR, Symptom Tracking and Reporting; QOL, quality of life; QLQ‐C30, Quality of Life Questionnaire‐Core 30; HSI, Hornheider Screening Instrument; MNA, Mini Nutritional Assessment; BPI, Brief Pain Inventory; PRO, patient‐reported outcome; PROMPT‐Care, Patient‐Reported Outcome Measures for Personalised Treatment and Care; SCNS‐ST9, Supportive Care Needs Surveying‐Screening Tool 9; HADS, Hospital Anxiety and Depression Scale

**Table 2:** Patient‐level barriers.

	Number of studies	Studies
Time required to complete PROMs	9	Hughes et al., 2004; Basch et al., 2005; Snyder et al., 2010; Snyder et al., 2013; Judson et la., 2013; Schepers et al., 2016; Trautmann et al., 2016; Duman‐Lubberding et al., 2017; Wang et al., 2018
Patient inability to complete PROMs	6	Snyder et al., 2010; Snyder et al., 2013; Judson et la., 2013 Baeksted et al., 2017; Girgis et al., 2017; Wang et al., 2018
Difficulty using electronic devices to complete PROMs	4	Basch et al., 2005; Snyder et al., 2013; Baeksted et al., 2017; Wang et al., 2018
Perceived irrelevance of PROMs and their lack of value	4	Kanatas et al., 2009; Snyder et al., 2010; Snyder et al., 2013; Trautmann et al., 2016; Girgis et al., 2017; Duman‐Lubberding et al., 2017;
Concerns that PROMs may compromise the HP to patient relationship	2	Snyder et al., 2010; Snyder et al., 2013
Concerns around privacy	1	Hughes et al., 2004

**Table 3:** HP‐level barriers.

	Number of studies	Studies
Insufficient time to interpret, action and discuss PRO data with patients during clinics	7	Hughes et al., 2004; Kanatas et al., 2009; Snyder et al., 2010; Daveson et al., 2012; Schepers et al., 2016; Girgis et al., 2017; Duman‐Lubberding et al., 2017
Lack of knowledge regarding how to interpret and integrate PROs into clinical practice	4	Kanatas et al., 2009; Daveson et al., 2012; Trautmann et al., 2016; Baeksted et al., 2017
Perceived uselessness of certain PRO data	3	Kanatas et al., 2009; Snyder et al., 2013; Girgis et al., 2017
Difficulty using the electronic PRO collection system	2	Baeksted et al., 2017; Girgis et al., 2017

**Table 4:** Service‐level barriers.

	Number of studies	Studies
Lack of integration of PRO into clinical workflows	5	Hubbard., 2014; Schepers et al., 2016; Trautmann et al., 2016; Baeksted et al., 2017; Duman‐Lubberding et al., 2017
Inability to action PRO data	3	Hughes et al., 2004; Basch et al., 2005; Girgis et al., 2017; Duman‐Lubberding et al., 2017
Inadequate information technology (IT) infrastructure to enable easy collection and use of PROs	3	Snyder et al., 2013; Schepers et al., 2016; Baeksted et al., 2017
Insufficient resources to implement PRO collection in clinics and refer patients to based on PRO data	2	Kanatas et al., 2009; Schepers et al., 2016

Patient‐level barriers to routine PRO use are outlined in Table 2. The most frequently reported barrier was time for patients to complete PROMs. Patient inability to complete PROMs, difficulty using electronic devices and perceived irrelevance of PROMs and their lack of value were other prominent barriers identified.

HP‐level barriers are outlined in Table 3. Major barriers included time, HPs’ lack of knowledge on how to interpret and integrate PROs into their clinical practice and HP difficulty using the electronic PRO collection system.

Service‐level barriers are outlined in Table 4. A prevalent barrier was the inability to integrate routine PRO use into clinical workflows. Other recurrent barriers included the lack of ability to action PRO data and inadequate information technology infrastructure to enable easy PRO collection.

## Discussion

Given its many potential benefits to patient care and outcomes, there is widespread interest in using PROMS to collect PROs to guide real‐time patient care. Implementing such practices however can be challenging. Barriers can exist at the patient, HP and service levels. These require recognition and analysis to understand how to best provide a supportive context for, and an effective approach to, facilitating PRO collection. This review sought to identify and elucidate prevalent barriers to routine PRO use in the oncology setting.

### Patient‐level barriers

The most frequent patient‐level barrier reported in the literature was the time for patients to complete PROMs. Due to the nature of their disease and treatment, cancer patients often undergo multiple time‐consuming appointments, procedures and tests. Thus, the time required to completed PROMs may be perceived negatively. In order to overcome this barrier, it is important to select the appropriate PROM that obtains the necessary data from patients but is not so long and complex that it becomes burdensome for them to complete.^22^

Another barrier that has been reported on in the literature is patients’ incapacity to complete PROMs, and this can be due to several reasons: disability or difficulty with reading and responding to the questionnaire measures; difficulty recalling their symptoms and remembering to complete PROMs; and being too unwell to report symptoms.^14^, ^27^, ^28^, ^32^, ^34^, ^35^ A solution to this barrier could be for reports from a proxy (e.g. spouse, significant other, caregiver) to be considered in circumstances where communication via proxy is the sole method of communication with patients.^36^ Electronic PRO collection often requires patients to self‐login and navigate through web portals which may prove challenging to those with limited technological experience. Thus, there is need for electronic PRO collection systems to be user‐friendly and for adequate support (e.g. education sessions, informative pamphlets) to be available to help patients complete PROMs.

Perceived irrelevance of PROMs and their lack of value was another barrier that has been identified. Indeed, multiple studies have revealed that patients felt that certain PROM questions were unrelated to them and their situation that they neither received adequate explanations about what PROs and PROMs were, nor understood the value of PRO collection.^10^, ^12^, ^14^ Therefore, efforts should be made to ensure that whenever PRO collection is planned that there is clarity around what the purpose of collection is, what the most appropriate PROM should be and that patients are well informed of these details.^17^ Lastly, further barriers such as patient concerns that PROM use may hinder their relationship with their HP can be mitigated by explaining to the patient that their routine clinical appointments and care will not be compromised whilst patient concerns around privacy can be addressed by using secure, password‐protected PRO collection systems.

### HP‐level barriers

The time required for HPs to educate patients on the value and use of PROs, administer PROMs and follow‐up on non‐responders may be considerable. Furthermore, it takes time for HPs to check, interpret and act meaningfully on the PRO data. Interestingly, although time has been highlighted in the literature as a barrier to PROM use, there are studies that have reported that PRO use does not take more time and indeed may save time in clinics.^17^, ^27^, ^37^ This is because HPs do not have to ask patients questions already screened by PROMs and instead focus on PROM detected issues. There is also the further positive benefit of deferring clinic appointments if patients report that they are not experiencing any problems. ^37^ Future research comparing HPs’ clinic times from initial PROM implementation, to when HPs are well attuned to using them, would be worthwhile.

HPs’ lack of knowledge on how to interpret and integrate PROs into their clinical practice was another important barrier. This highlights the importance of incorporating staff training, education sessions and guidelines into the early stages of PRO implementation to help HPs effectively use PRO data in their clinical practice. ^25^, ^38^, ^39^ Unlike in the research (clinical trials) setting where PROs from an intention group of patients is compared to those from a control group of patients, in the setting where routine PROM data are intended to be used to guide real‐time clinical care, there are several considerations that need to be addressed. Clarity on the purpose of the PROM data and the need for threshold values of PROM scores (i.e. actionable triggers) are important. Without these, it is not possible for HPs to meaningfully intervene and establish clinical pathways to assist patients with the concerns identified. There is a need for future work focusing on identifying actionable thresholds for specific items on existing PROMs. HP perceived uselessness of certain collected PRO data was another barrier to PROM use. Studies revealed that some PROM questionnaires highlighted issues already known to the clinical team or provided little value to the clinical management of patients. ^14^, ^31^, ^34^ This reiterates the importance of selecting relevant PROMs for specific patient populations and having streamline departmental‐specific processes in place such that the collected PRO data may be effectively applied into clinical practice.

Lastly, a notable HP‐level barrier that emerged from this review was HP difficulty using electronic PRO collection systems. HPs found it difficult to navigate multiple log ins, especially as often, third party software was required for PRO collection and interpretation of PRO data. ^14^, ^27^ Therefore, in addition to essential staff training, a clear, user‐friendly interface to collect and display PRO data (e.g. using graphics, dashboards, threshold lines, colour codes) would improve engagement and help HPs interpret PROs and act on them. ^9^, ^40^

### Service‐level barriers

A common service‐level barrier was the difficulty or inability to integrate routine PRO use into existing clinical workflows. Giving patients the flexibility to complete PROMs on their own devices and in their own time can be one solution to this problem and may relieve time pressures in the clinic setting. Establishing clinical pathways that can be automatically activated based on PROM scores may also be of potential benefit. For example, patients who score above set anxiety or distress thresholds may be automatically referred to psychosocial services whilst patients with increased baseline pain scores can be examined for the source of their increased pain, provided with suitable pain medications and referred to other HPs.^14^, ^16^, ^17^, ^18^ However, certain cases may be practically challenging. Patients may have issues which cannot be effectively resolved by available services and referrals to appropriate services may not result in timely attention to patient issues due to resource limitations; these, in turn, may cause additional concerns related to duty of care and liability.^12^, ^14^, ^29^, ^30^ In fact, appropriate and adequate staffing levels and referral networks are essential if patients’ concerns are to be attended to in a timely manner. Aside from the benefit at the individual patient level, the process of identifying clinical pathways to action PRO data can be used at a cohort level within health services to explicitly identify gaps in their service.

Inadequate information technology (IT) infrastructure for easy PRO collection and use was another prominent barrier at the service level. A major issue is the non‐integration of PRO data into hospital electronic medical records (eMR). In cases where the existing hospital eMR cannot support PRO collection, HPs need to log into multiple systems which carries the risk of poor care coordination, inefficiencies in activating clinical pathways and missed opportunities for improved care.^41^, ^42^, ^43^ Therefore, efforts are necessary to incorporate PROM collection capability into the existing cancer eMR systems. Finally, adequate resources and staffing infrastructure to carry out activities such as administering PRO collection, helping patients fill out PROMs on paper, tablets or kiosks and operate as well as trouble shoot the PRO collection system would be crucial considerations.

## Limitations

The routine use of PROMs to guide real‐time patient care is still in its infancy. As such, studies discussing the barriers to this practice are limited in number and involve small samples. Therefore, this review may not have exhaustively captured all the potential barriers to the routine use of PROMs for PRO data collection. Furthermore, as the characteristics of PRO data collection (e.g. frequency, PROM tools, patient population and the clinical environment) varied amongst the papers reviewed, the barriers outlined in this review may not apply widely to all oncology settings and may not have captured perspectives of all oncology HPs. involved in the delivery of patient care. For example, radiation therapists who are involved in the daily care of patients receiving radiation therapy over many weeks (as long as 7 weeks for some curative courses of radiation therapy) are under‐represented in studies despite their unique position, due to daily contact with patients, to facilitate early detection of issues during treatment. Ideally, future studies will address this omission and include radiation therapists’ perceptions of barriers, and indeed facilitators, to routine PROM use in cancer care.

## Conclusion

This review examined potential barriers to routine PROM use in the oncology context. At the patient level, time, incapacity and difficulty using electronic devices were prominent barriers. At the HP level, major barriers included lack of time and knowledge to interpret and integrate PRO data into individual patient care pathways. Lastly, notable barriers at the service level included difficulty integrating PROM collection into routine clinical workflows and inadequate IT infrastructures to facilitate PRO collection and integration into the eMR. It is recommended that individual services independently examine the barriers relevant to their patient population, HPs and health service and address these as part of any PROM collection implementation initiatives.

## Conflict of Interest

The authors declare no conflict of interest.
